# Cincinnati Dental Association

**Published:** 1873-08

**Authors:** D. C. Hawxhurst


### CINCINNATI DENTAL ASSOCIATION.

Monday Evening, June 9, 1873.

The Cincinnati Dental Association met in the evening, Dr H. R. Smith occupying the chair. The committee appointed to investigate the question of prices, made a provisional report and asked for more time.

A report was called for from the Microscopical Section, as regards work done, and proposed to be done. Here, too, no final report could be given. Dr. Hawxhurst stated that there had been consideration given to the subject, and that, at Dr. Taft's suggestion, he had written down a plan of study, which in the absence of other important business, he was requested to present.

He said that in working out a course of microscopical study for a body of dental practitioners, it appears to be the better way to choose some restricted line of investigation. To study the whole of animate and inanimate nature, would be too great a task. It is better to leave out of consideration all substances except living tissues, and it would even here be well to restrict ourselves to the study of only some particular tissues, which may be of the greatest interest to the dental practitioner. But here the field is vast, and we may as well begin with some particular tissue. The teeth offer points of much interest and are likely to be especially attractive.

If we begin with dentine, which constitutes the body of the tooth, and carry our investigations onward through the enamel and cementum, and then proceed to the study of the associate parts, we shall have adopted a very good order.

Dentine may be studied as a normal tissue, until all its natural characteristics are made out, and until we understand pretty well what it is when healthy, and naturally constituted. Its anomalies may then be taken up, and all the variations of form which it can undergo without taking on pathological conditions, may be presented. We may then proceed to study all the numerous modifications of this tissue, which are produced by disease.

This same three-fold study may be carried into our investigation of enamel; taking up first its normal varieties, then its anomalous characters, and finally the differences of structure that pathology may introduce into it.

After proceeding to the study of cementum and investigating according to the same plan, we shall be tolerably well acquainted with the microscopical characters of the teeth and may then lay out accurately a further course of study.

There was much discussion upon this report, and upon other questions that were introduced by an essay that was read upon the subject of Lime Salts.

Dr. Hawxhurst tendered his resignation as Secretary of the Association, and Dr. S. E. Goe was elected in his place.

By vote of the Association the initiation fees and dues of Drs. Goe and Hawxhurst were remitted in consideration of their past and prospective services.

The Association adjourued to meet on the 2nd Tuesday of July, at the office of Dr. A. Berry.

D. C. HAWXHURST. Sec'y

